# Discordant transmission of bacteria and viruses from mothers to babies at birth

**DOI:** 10.1186/s40168-019-0766-7

**Published:** 2019-12-10

**Authors:** Rabia Maqsood, Rachel Rodgers, Cynthia Rodriguez, Scott A. Handley, I. Malick Ndao, Phillip I. Tarr, Barbara B. Warner, Efrem S. Lim, Lori R. Holtz

**Affiliations:** 1 School of Life Sciences, Arizona State University, Tempe, AZ 85287 USA; 2 Center for Fundamental and Applied Microbiomics, The Biodesign Institute, Tempe, AZ 85287 USA; 3 Department of Pediatrics, Washington University School of Medicine, St. Louis, MO 63110 USA; 4 Department of Pathology & Immunology, Washington University School of Medicine, St. Louis, MO 63110 USA; 5 Department of Molecular Microbiology, Washington University School of Medicine, St. Louis, MO 63110 USA

**Keywords:** Virome, Microbiome, Transmission

## Abstract

**Background:**

The earliest microbial colonizers of the human gut can have life-long consequences for their hosts. Precisely how the neonatal gut bacterial microbiome and virome are initially populated is not well understood. To better understand how the maternal gut microbiome influences acquisition of the infant gut microbiome, we studied the early life bacterial microbiomes and viromes of 28 infant twin pairs and their mothers.

**Results:**

Infant bacterial and viral communities more closely resemble those of their related co-twin than unrelated infants. We found that 63% of an infant’s bacterial microbiome can be traced to their mother’s gut microbiota. In contrast, only 15% of their viral communities are acquired from their mother. Delivery route did not determine how much of the bacterial microbiome or virome was shared from mother to infant. However, bacteria-bacteriophage interactions were altered by delivery route.

**Conclusions:**

The maternal gut microbiome significantly influences infant gut microbiome acquisition. Vertical transmission of the bacterial microbiome is substantially higher compared to vertical transmission of the virome. However, the degree of similarity between the maternal and infant gut bacterial microbiome and virome did not vary by delivery route. The greater similarity of the bacterial microbiome and virome between twin pairs than unrelated twins may reflect a shared environmental exposure. Thus, differences of the inter-generation transmissibility at birth between the major kingdoms of microbes indicate that the foundation of these microbial communities are shaped by different rules.

Video Abstract

## Background

The gut undergoes a profound ecological transition as the infant leaves the near sterile or sterile womb and becomes home to diverse microbial populations. This once in a lifetime event has lasting effects on the infant by shaping growth [1], conferring resistance to infection [2], calibrating inflammation [3], and programming immune function [4]. Remarkably, however, we know very little about how and when the neonatal gut bacterial microbiome and virome are acquired.

Histological evidence [5] and sequencing data [6, 7] suggest that the infant might encounter microbes in the womb, thereby starting colonization in that venue. However, several recent publications find no convincing evidence of bacterial or viral nucleic acid in human amniotic fluid or placenta [8–11], calling into question the in utero colonization hypothesis. Several studies have demonstrated striking differences between the maternal and infant gut bacterial microbiome communities, but also provide evidence of shared bacterial species among related mothers and infants [12–15].

During the first days of life, portions of the infant gut microbiome can be traced to multiple maternal sources including the vaginal, skin, oral, and gut microbiomes. However, of these sources, colonization by bacteria that are found in the mothers’ gut is the predominant driver of long-term persistence [16]. Indeed, seeding by vertically transmitted bacteria (such as *Bifidobacterium, Ruminococcus, Coprococcus* species) causes transcriptionally active colonization in the infant gut [13]. Various factors such as birth route and food source may affect transmission to her infant. The effect of birth route on the similarity of newborn and maternal bacterial gut microbiome composition is conflicted [17, 18]. While diet is known to impact bacterial microbiome composition over time, interestingly the bacterial gut microbiomes of breastfed vaginally delivered newborns do not differ significantly from those receiving formula [17, 18].

Considerably less is known about early colonization of the infant gut with viruses and if vertical transmission plays a role [19]. Previously, we described a high diversity of bacteriophages in stools from infants during their first 96 h of life [20]. The source of these bacteriophages is unknown; our data suggest mother-to-infant transmission could play a significant role. For instance, vertical transmission of bifidobacteria bacteriophages can be traced to breastmilk [21]. Further, at 1 year of age, vaginally delivered infants have increased gut viral diversity than infants delivered by C-section [22], suggesting that other factors may also influence infant virome acquisition.

Here, we sought to understand how microbes initially colonize the human infant gut. To do so, we ask if earliest in life stools (obtained at 3–93 h of age) from 28 term twin pairs share their mothers’ stool virome and bacterial microbiome. This cohort additionally offers a unique opportunity to determine the consistency of vertical transmission for co-twins. Such data will greatly increase our understanding of how microbes initially occupy the infant gut.

## Results

### Gut bacterial microbiome of infant twin pairs and their mothers

**Fig. 1:**
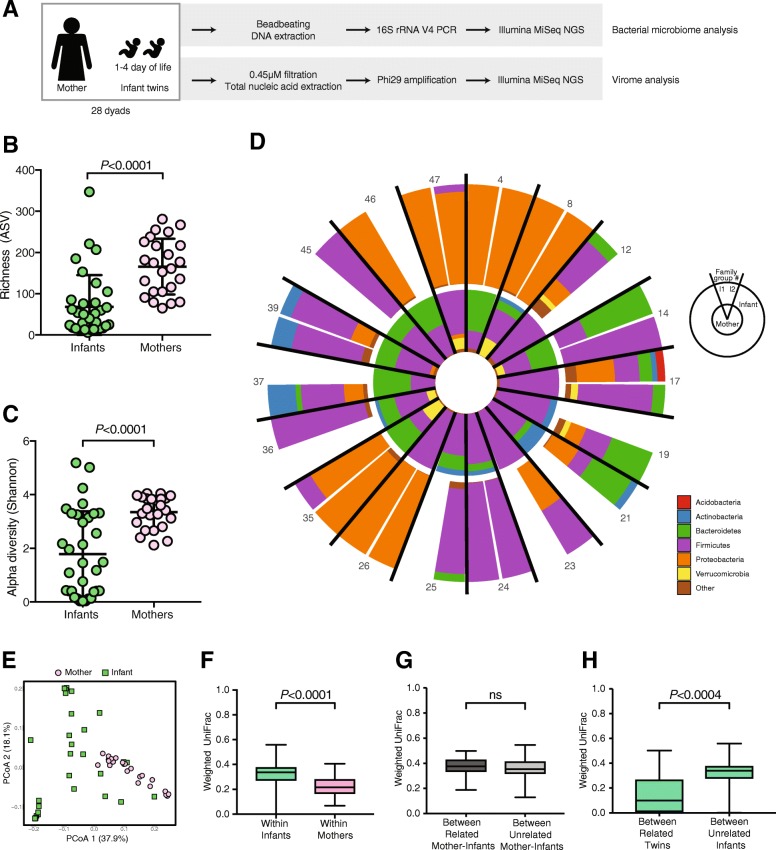
Bacterial microbiota analysis of mothers and infants. **a** Overview of study design. **b** Richness of bacterial amplicon sequence variants (ASV) in infants and mothers. Statistical significance assessed by Mann-Whitney test. **c** Alpha diversity of bacterial ASV in mothers and infants. Statistical significance assessed by Mann-Whitney test. **d** Relative abundance of bacteria at phylum level for mothers and infants; I1 (infant twin 1) and I2 (infant twin 2). **e** PCoA plot of weighted UniFrac distances. **f** Weighted UniFrac pairwise comparison within infants and within mothers. Statistical significance assessed by Mann-Whitney test. **g** Weighted UniFrac pairwise comparison between related mother-infant (*n* = 27 pairs) and between unrelated mother-infant (*n* = 765 pairs). Statistical significance assessed by Mann-Whitney test. **h** Weighted UniFrac pairwise comparison between co-twins (*n* = 11 twin pairs) and between unrelated infants (*n* = 517 pairs). Statistical significance assessed by Mann-Whitney test

We performed 16S rRNA gene sequencing of stools from 56 infants (28 twin pairs, collected on or before the fourth day of life) and their 28 mothers (Fig. 1a). Of the 84 samples, five samples failed to amplify, four were excluded due to low (< 1000) read counts, and 18 generated sequences indistinguishable from buffer control samples (see the “Methods”) and were also excluded from analysis (Additional file 6: Table S1). Sequences from the remaining 24 maternal and 33 infant samples correspond to 2615 different bacterial taxa. Both richness and Shannon diversity of infant bacterial gut communities were significantly less than in their mothers’ stools (both *p* < 0.001) (Fig. 1b and c). Proteobacteria dominate several infant bacterial communities, but were much less abundant in the maternal samples (Fig. 1d). Principal coordinate analysis (PCoA) of weighted UniFrac distances demonstrate distinct and tight clustering of those from maternal samples and a more diffuse distribution of infant samples (Fig. 1e). Group significance testing with ADONIS confirmed that the clustering of samples by source (mother or infant) was statistically significant and accounts for approximately 23% of the variation in weighted UniFrac distances (*p* < 0.001, *R*^2^ = 0.2347). To determine if other factors might be associated with specific microbial communities, we performed a multivariate analyses and random forest classification; both methods demonstrate that mothers and infants have significant differences between their bacterial microbiome (Additional file 1: Figure S1B–E). Race was identified as a significant determinant of bacterial community composition by multivariate analysis, but not by random forest (Additional file 1: Figure S1B). Therefore, age of host (i.e., adult vs. infant) was the only factor that separated the bacterial communities in this cohort.

Infant bacterial communities and the relationship to maternal communities were further characterized by pairwise weighted UniFrac distances. Similar to the PCoA data (Fig. 1e), the pairwise distance between all infant bacterial communities was significantly greater (*p* < 0.001) than the mean distance between all maternal communities (Fig. 1f). To test if the infant gut bacterial communities more closely resembled those of their own mother than all unrelated mothers, we measured the distances of infant bacterial community structure to their cognate mother versus the distances to all mothers in the study. Interestingly, the distance of an infant to their own mother was no different than to an unrelated mother (Fig. 1g). However, the mean distance between co-twin bacterial communities was significantly less (*p* < 0.001) than to unrelated infants (Fig. 1h). The distance between co-twins did not differ with zygosity (Additional file 1: Figure S1F), suggesting that age and environmental exposures are the more important determinants of the bacterial communities.

**Fig. 2:**
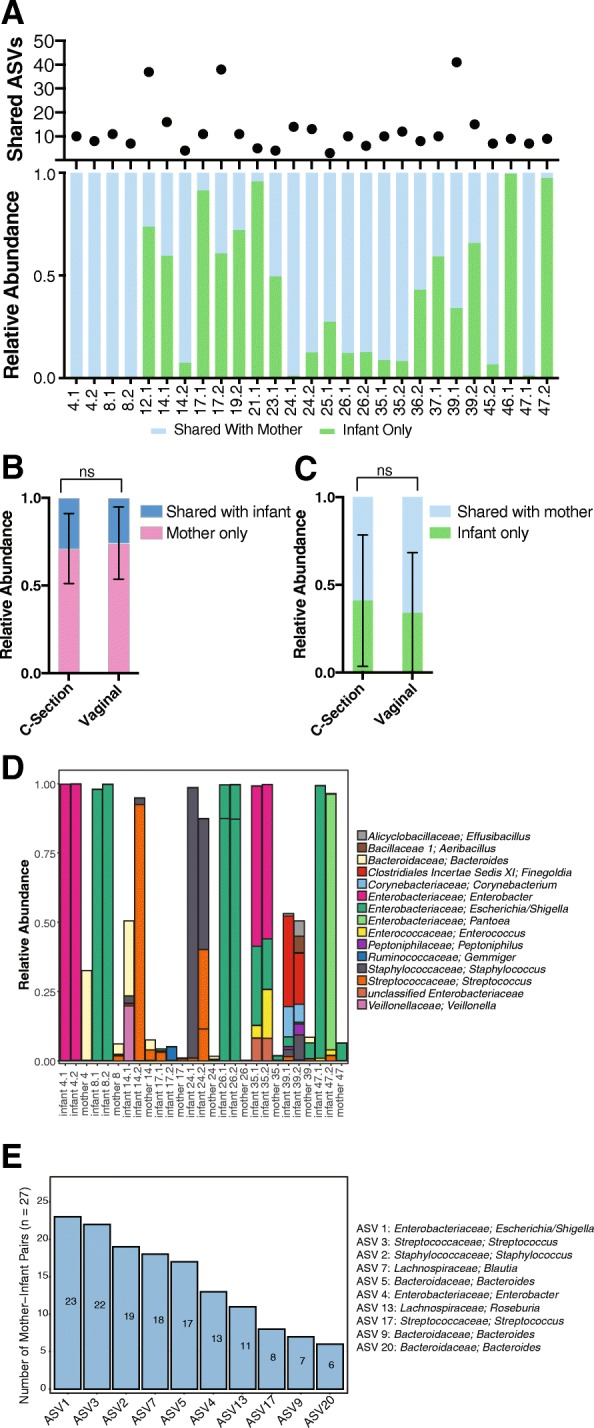
Bacterial ASV transmission analysis. **a** The number of ASVs shared between mother and infant and the relative abundance of the infant bacterial microbiome that is shared with mother. **b** Average relative abundance of maternal bacterial microbiome that is shared with infant by delivery route. Statistical significance assessed by Mann-Whitney test. **c** Average relative abundance of infant bacterial microbiome that is shared with mother by delivery route. Statistical significance assessed by Mann-Whitney test. **d** Relative abundance of ASVs present in both twin pairs (> 0.05). Taxonomy shown at the genus level. **e** Frequency plot of the 10 most commonly transmitted ASVs

To develop a more detailed view of the relationship of related mother-infant bacterial communities, we identified bacterial taxa shared between the communities of mothers and their related infant(s). On average, a related mother and infant share 12 bacterial taxa which accounted for 63% of the total reads in the infant’s sample (Fig. 2a). We then asked if mothers share differing amounts of their bacterial communities with their infants depending on the route of delivery. The average relative abundance of reads from shared taxa within the mother did not vary by route of delivery (Fig. 2b). We next examined if the amount of the infants’ bacterial microbiome that was shared with their mother differed when comparing infants born vaginally versus by C-section and found no influence of route of delivery (Fig. 2c). We next asked what bacterial taxa were shared between twins and what is the relative of abundance of these bacterial taxa in both the infant and maternal stools. In general, while the bacterial taxa present in both twins had a high relative abundance in the infant, they represented only a small fraction of the maternal bacterial community (Fig. 2d and Additional file 7: Table S2). We additionally examined if there were ASVs which were frequently shared by mother-infant pairs across all the families (Fig. 2e). One ASV belonging to the Escherichia/Shigella genus was shared in 23 of the 27 mother-infant pairs (Additional file 8: Table S3). The relative abundance of these 10 commonly shared ASVs was again generally less in the maternal samples than the infant samples (Additional file 2: Figure S2). These data collectively indicate that the mother shares a relatively small proportion of bacterial community with her infants, that these shared taxa make significant contributions to the infant’s bacterial microbiome, and that the amount either shared (mother) or received (infant) does not vary by delivery route.

### Gut virome of infant twin pairs and their mothers

**Fig. 3:**
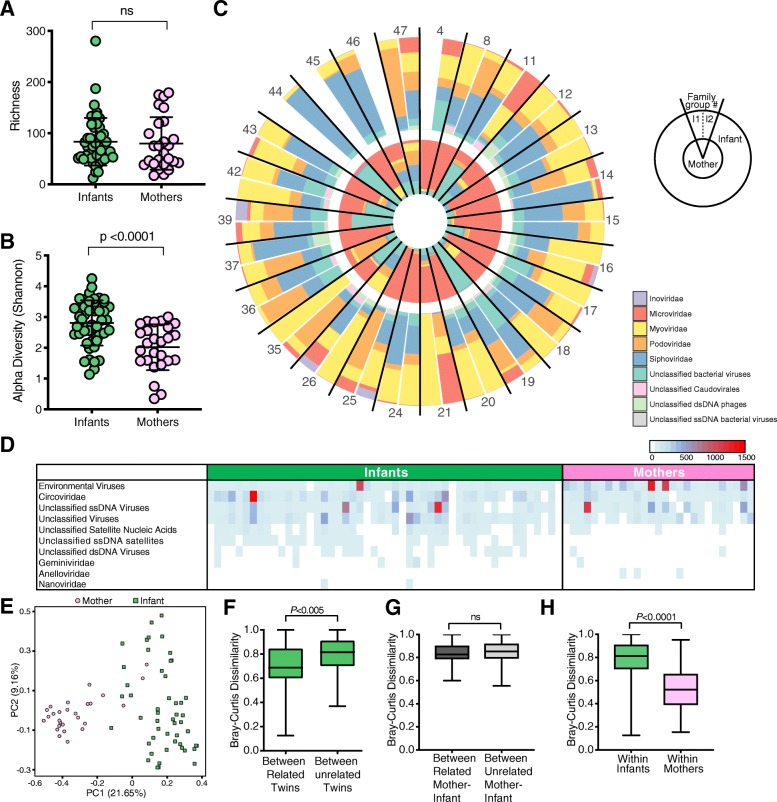
Virome analysis of mothers and infants. **a** Richness of viral species in infants and mothers. Statistical significance assessed by Mann-Whitney test. **b** Alpha diversity of viral species in mothers and infants using Shannon index. Statistical significance assessed by Mann-Whitney test. **c** Relative abundance of phages at family level for mothers and related infants within each family. I1 (infant twin 1) and I2 (infant twin 2). **d** Heatmap of eukaryotic families and unclassified categories for infants and mothers. **e** PCoA plot using Bray-Curtis distance. Color represent category of samples as infants or mothers. **f** Bray-Curtis distance for viral species between related twins and between unrelated twins. Statistical significance assessed by Mann-Whitney test. **g** Bray-Curtis distance for viral species between related mother-infant and between unrelated mother-infant. Statistical significance assessed by Mann-Whitney test. **h** Bray-Curtis distance for viral species within infants and within mothers. Statistical significance assessed by Mann-Whitney test

To assess early life mother-infant virome transmission, we performed metagenomic sequencing of the same fecal specimens from 56 healthy infants (28 twin pairs, collected on or before the fourth day of life) and their 28 healthy mothers. Seven of the 84 samples were not entered into analysis: five did not amplify, one had insufficient volume after filtering, and one had low sequencing reads (3768) (Additional file 6: Table S1). This resulted in a virome dataset for 50 infants and 27 mothers, i.e., 26 families with mother and at least one infant. There was no significant difference between the sequence depth of infants (mean 131,846 ± 105,022) and maternal samples (mean 103,040 ± 39,986) (*p* = 0.271) (Additional file 3: Figure S3A). The mean infant and maternal gut virome species richness were similar (83 vs 78 species, respectively) (*P =* 0.6091) (Fig. 3a), but the gut virome Shannon diversity was significantly greater in infants than in mothers (*p* < 0.0001) (Fig. 3b). The most abundant bacteriophages in the maternal gut virome were from the *Microviridae* family, whereas infant gut viromes consisted predominantly of bacteriophages from the *Myoviridae*, *Podoviridae*, and *Siphoviridae* families (Fig. 3c). Circoviruses (*Circoviridae* family) and unclassified viruses (within the “ssDNA viruses” and “environmental viruses” taxonomic group classifications) were frequently detected in both mother and infant samples (Fig. 3d). Taken together, these data suggest considerable age-dependent virome differences (i.e., whether the specimen is from an adult or infant) that might confound assessments of familial relationships between related mother-infant pairs.

To resolve this, we first compared virome diversity by measuring the unweighted Bray-Curtis distance between gut virome communities. PCoA indicated that the maternal gut viromes differ from infant gut viromes (Fig. 3e). To further understand additional factors that might influence the composition of the infant gut virome, we performed multivariate analyses of the cohort metadata (e.g., delivery route, feeding type, delivery site) and found that age (i.e., maternal or infant origin of stool) was significantly associated with differences in the virome (Additional file 3: Figures S3B–D). This was corroborated by building classifier models with random forest that accurately distinguished the virome of mothers from infants (AUC = 0.996) (Additional file 3: Figure S3E). Maternal BMI (pre-pregnancy) was also identified as a determinant by the multivariate analyses but was not a significant factor in random forest (Additional file 3: Figure S3B).

Because mother-infant dyads shared a substantial subset of their bacterial microbiome, we hypothesized that they would also harbor relatively similar viromes. Consistent with this hypothesis, we found that related infants shared a more similar gut virome to their co-twin than to unrelated infants (Fig. 3f, *p* < 0.005). This co-twin virome similarity was not influenced by their zygosity (Additional file 3: Figure S3F). However, infant gut viromes were highly dissimilar from their own mother. In fact, the ecological distance between an infant and their own mother was the same as it was to unrelated mothers (Fig. 3g, *p* = 0.1400). This might be explained in part by the significantly lower virome interpersonal variation among mothers as compared to infants (Fig. 3h, *p* < 0.0001).

**Fig. 4:**
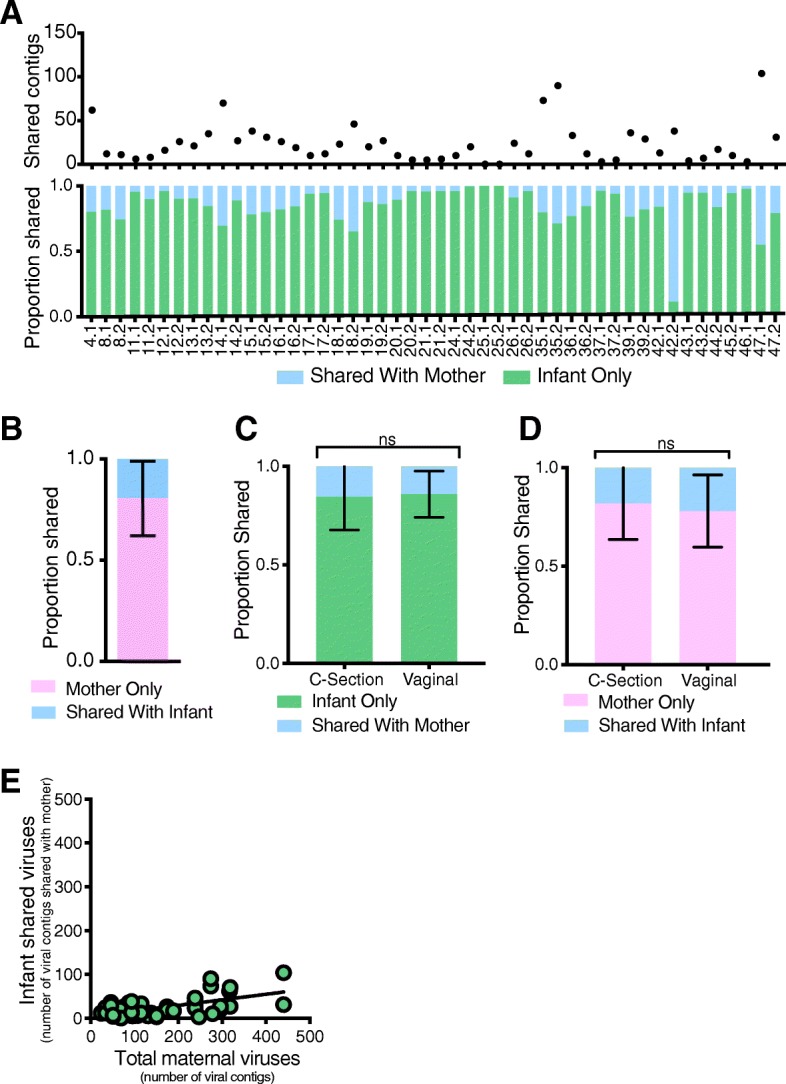
Viral contig transmission analysis. **a** The number of contigs shared between mother and infant and the proportion of infant virome that is shared with mother or is infant only. **b** Average contig proportion of mother virome that is shared with infant or is mother only. **c** Average contig proportion of infant virome that is shared with mother by delivery route. Statistical significance assessed by Mann-Whitney test. **d** Average contig proportion of maternal virome that is shared with infant by delivery route. Statistical significance assessed by Mann-Whitney test. **e** Plot of the number of infant shared viral contigs vs. the total number of maternal contigs. Linear regression line fit to data

These diversity measurements do not account for differences in virome abundance (i.e., unweighted distances). Hence, we assessed virome transmission in terms of the shared viral abundance. For example, a large proportion of the infant virome might be established from transmission of a relatively small number of viruses from the mother. To quantify the proportion of the infant virome shared with their mother, we mapped the infants’ sequencing reads to viral contigs assembled from the infant(s) and their mothers’ virome. On average, only 15.0% of the viruses present in the infant stool could be mapped back to the sequences in their mother’s stool (Fig. 4a). Conversely, 19.8% of the mother’s virome was shared with her infant (Fig. 4b). The proportion of the viruses shared between mothers and their infants was not influenced by delivery route (Fig. 4c and d). Further, the number of viruses transmitted from mother-to-infant was independent of the number of viruses in the mother’s virome (Fig. 4e, slope *m* = 0.127). Thus, unlike the bacterial microbiome, infants shared only a limited proportion (approximately 15%) of their gut virome with their mother. These results were also validated by analyses of bacteriophage contigs identified by VirSorter (Additional file 4: Figure S4).

### Route of delivery influences transkingdom interactions between bacteria and bacteriophage

**Fig. 5:**
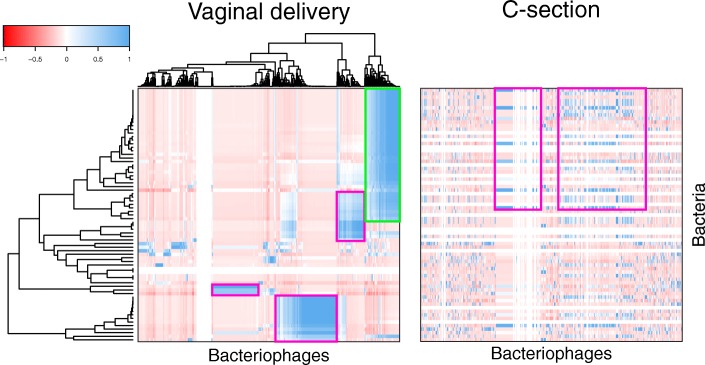
Transkingdom interaction between bacteria and bacteriophage. Correlation of bacterial ASVs and viral contigs shared between infants and mothers. Heatmap shows the Pearson correlation between bacterial ASVs and bacteriophage contigs in infants from vaginal delivery (left) clustered by hierarchical clustering. Correlations from infants delivered by C-section (right) were clustered in the same order as correlations from infants delivered vaginally. Examples of differences in transkingdom interactions between infant delivered vaginally vs. C-section are outlined in pink and green

After demonstrating that only a subset of the virome and bacterial microbiome is shared between mothers and infants, we sought clinical factors that might influence their transkingdom interactions. Hierarchical clustering indicated that the gut bacterial microbiomes of infants born by vaginal delivery (*Ruminococcaceae*, *Lachnospiraceae*, *Bacteroidales*, and *Lactobacillales* amplicon sequence variants (ASV)) were strongly correlated with bacteriophages of the *Microviridae*, *Myoviridae*, *Siphoviridae*, and *Podoviridae* families (Fig. 5). In contrast, microbiome interactions of infants born by C-section delivery were discordant from infants born by vaginal delivery. First, bacteriophages also detected in the stools of infants born by C-section correlated with different bacteria than the same bacteriophages from infants delivered vaginally. In children born by C-section, *Microviridae*, *Myoviridae*, *Siphoviridae*, and *Podoviridae* bacteriophages were strongly correlated with *Staphylococcaceae*, *Bacteroidaceae*, *Lachnospiraceae*, and other *Ruminococcaceae* ASVs. For example, Parabacteroides phage YZ-2015b from *Microviridae* family correlated with *Bacteroidales* ASVs (*Bacteroides uniformis* and *Parabacteroides distasonis*) in infants born through vaginal delivery, but correlated with other *Bacteriodales* ASVs (*Bacteriodes ovatus*) in infants born through C-section (Fig. 5, compare pink outline; Additional file 5: Figure S5A). Second, microbiome interactions in stool were also absent from infants born by C-section compared to those born vaginally (Fig. 5, compare green outline), indicating a transkingdom signature which was unique to each delivery route. This was corroborated by Pearson’s correlations between bacterial ASVs- and VirSorter-identified contigs (Additional file 5: Figure S5B). Therefore, this suggests that delivery route might influence interactions between the bacterial microbiome and virome.

## Discussion

The dynamic neonatal bacterial microbiome and virome likely influence or even define the more stable adult bacterial microbiome and virome. It is thought that proper assembly of these communities promotes health. However, precisely how the neonatal gut bacterial microbiome and virome are initially populated is not well understood. Here, we find that twins and their mothers have distinct gut virome and bacterial microbiomes. This finding is consistent with studies showing bacterial microbiome and virome composition is age dependent [23, 24]. However, despite having unique communities, mothers and their infants do share some components, predominantly among the bacterial communities. Interestingly the proportion and specific taxa that are shared differed within and between families. It is important to note, that while the proportion of the bacterial microbiome that a mother shares with her infant is relatively small, these bacteria do account for over half of the infant’s bacterial microbiome. In our study, the average of maternal-matched bacterial microbiome is 63%, similar to the ranges (30–70%) of other cohorts [16, 18, 25, 26]. We do note that 18 infants stool bacterial microbiomes were indistinguishable from buffer background, and if we interpret these as having no maternal transmission, 63% may be an overestimate. Addtionally, 16S rRNA gene sequencing may not always have sufficient resolution to distinguish different strains which would also lead to an overestimation of shared bacteria. However, together, these studies emphasize that the maternal gut microbiome can be a substantial, though not the exclusive, source of an infant’s early bacterial microbiome.

Our virome data are unique, and offer substantial contrast to the bacterial microbiome. Our finding of only 15% of the infant’s virome in the cognate newborn’s mother’s stool strongly suggest that infant gut viruses are likely from other habitats (e.g., mother’s skin, breastmilk) or the inanimate environment. Second, it is possible that the early window in this study (1–4 days of life) is when bacterial colonization is still underway. This builds on the longitudinal study of Ferretti et al., which demonstrated that maternal gut-sourced bacteria increasingly dominate the infant microbiome by 4 months [16]. In this model, it is plausible that “maternally sourced” viruses that can replicate in “maternally sourced” bacteria would become enriched weeks to months after bacterial colonization. This would be consistent with the shift in bacteriophage community previously reported to occur from 0 to 2 years [20].

This twin study also offers unique data to compare vertical transmission and co-twin relationships. The greater similarity of the bacterial microbiome and virome between twin pairs than unrelated twins may reflect commonality of a shared environmental exposure. Shared exposure from a maternal site other than stool, such as maternal breastmilk, might be another source. Although breastfeeding did not significantly influence mother-to-infant bacterial transmission, the viromes of breastfed infants are more similar to their mother than were those of formula fed infants (Additional file 1: Figure S1G and Additional file 3: Figure S3G). Given the limitations of this cohort, future studies will be needed to clarify the role of diet. Nonetheless, this highlights that the dynamics of the acquisition of the human bacterial microbiome and virome clearly differ.

The neonatal gut virome consists of largely bacteriophage with only a small portion being eukaryotic viruses. Of note, we focused on the DNA virome as our previous study of the infant virome [20] (those four twin pairs are also included here) found a sparse RNA virus component early in life. As with the neonatal bacterial microbiome, the virome varies across individuals. In contrast to what has been described in adult twins, bacteriophage diversity does not correlate with bacterial diversity [27]. Additionally, the discrepancy in proportion of overlap between the maternal and infant virome and bacterial microbiome suggests that bacterial microbiome and virome are acquired from different sources.

Previous studies of the effect of birth route on the newborn gut bacterial microbiome have offered conflicting findings. While we found bacterial communities differed significantly between infants born vaginally versus those born by C-section, we were unable to find discriminating ASVs for delivery route. The shared proportion of the infant and maternal bacterial microbiome is not significantly different by delivery route. Furthermore, we found no difference between the Bray-Curtis dissimilarity of infant gut virome by delivery route, and we found no viral contig that discriminated between infants born vaginally versus by C-Section. Additionally, the proportion of infant and maternal virome that is shared is not significantly different between infants born vaginally versus by C-section. However, when we examined transkingdom interactions, we found that this relationship did differ by delivery route. This transkingdom interaction might reconcile why some studies show delivery route is an important factor, while others do not.

## Conclusions

In summary, our data demonstrates that while mothers and infants do share some of their gut microbial communities, the degree of sharing is more substantial for bacteria than for virus. Additionally, the proportion and specific taxa shared varies between and even within families. Lastly, bacterial-bacteriophage interactions differed depending on delivery route. Therefore, our data depict that the inter-generation transmissibility at birth diverges between the major kingdoms of microbes suggesting that the founding sources of these communities may differ.

## Methods

### Subjects

This study was approved by the Human Research Protection Office of Washington University School of Medicine in St. Louis and Missouri-Baptist Medical Center institutional review board. We obtained written informed consent from women pregnant with twin gestations were consented to collect stool specimens around the time of birth from the mother and her twins. Stools from the mothers were collected at home or in the hospital; infant stools were collected in the hospital. Home-produced samples were couriered to the laboratory in insulated envelopes containing frozen (− 20 °C) freezer packs and stored at − 80 °C until analysis. Twenty-eight families were selected in which maternal stools were collected peripartum and there was adequate sample from early in life from the twins. Metadata collected included mode of delivery, feeding content, maternal age, maternal weight and weight gain, and maternal race (Table 1 and Additional file 6: Table S1). This birth cohort has been described in various detail in prior publications [20, 23, 28–33].

**Table 1:** Cohort demographics

Infant age at time of stool (h) (median, IQR, range)	37.2 (16.7, 54.4), 3.3 to 92.5
Maternal stool time from birth (h) (median, IQR, range)	108 (36, 390), − 360 to 1680
C-section no. (%)	35 (62.5%)
Feeding (between birth and sampling) no. (%)	
Breastmilk	8 (14.3%)
Formula	16 (28.6%)
Mix	32 (57.1%)
Monozygotic no. (%)	30 (53.6%)

### Bacterial 16S rRNA gene sequencing

One hundred milligrams of stool was disrupted by bead beating and DNA was extracted using QIAamp DNA Stool Mini Kit on a QIAcube automated DNA extraction unit. In parallel, four buffer-only controls were disrupted by bead beating and similarly extracted to serve as extraction negative controls. PCR was performed using Golay-barcoded primers specific for the V4 region (F515/R806). Reactions were held at 94 °C for 2 min to denature the DNA, with amplification proceeding for 40 cycles at 94 °C for 15 s, 50 °C for 30 s, and 68 °C for 30 s; a final extension of 2 min at 68 °C. Stool and buffer samples, as well as 4 water negative controls, were amplified in triplicate, combined, and cleaned using Agencourt Ampure XP beads (Beckman-Coulter). Equimolar libraries were pooled and sequenced using an Illumina MiSeq sequencer (2 × 250 v2 kit) at the Center for Genome Sciences & Systems Biology at Washington University.

### Bacterial 16S rRNA gene analysis

16S rRNA amplicon sequences were demultiplexed using QIIME (Quantitative Insights Into Microbial Ecology, v 1.8.0) [34]. Read quality control and the resolution of amplicon sequence variants (ASVs) using the forward-reads only was performed with the DADA2 R package [35]. Taxonomy was assigned to the ASVs from the DADA2-formatted training files derived from the Ribosomal Database Project’s Training Set 16 [36]. ASVs were aligned using an R implementation of MSA [37] and arranged into a maximum likelihood phylogeny (GTR model with optimization of the proportion of invariable sites and the gamma rate parameter) using the phangorn package [38]. The ASV counts, taxonomy assignments, phylogeny, and sample metadata were combined to generate a PhyloSeq object for further analyses [39]. Five samples failed to amplify. Four samples with less than 1000 reads were excluded from analysis. To compare the bacterial community structure between the stool samples and controls we generated a multidimensional scaling (MDS) plot of the unweighted UniFrac distance with normal confidence ellipses. Eighteen samples encapsulated by the confidence ellipse of the buffer control samples were removed from further analysis (Additional file 1: Figure S1A). The Decontam R package “prevalence” method at a threshold of 0.25 was used to identify and remove remaining contaminating sequences [40]. Further ecological analyses were performed in R.

If a mother and one of her related infants had an identical ASV, then it was assumed to be shared between mother and infant; otherwise, it was exclusive to mothers or infants. Only families that had a sample from mother and at least one of her infants were used to determine potential mother to infant transmission.

### Virome sequencing

Fecal specimens (approximately 100 mg) were diluted 1:6 with phosphate-buffered saline (PBS) and filtered through a 0.45-μm-pore-size membrane. Total nucleic acid was extracted from the filtrate using COBAS Ampliprep (Roche). In parallel, PBS was filtered and extracted to serve as extraction reagent only control. Total nucleic acid from stool and negative controls was amplified with Phi29 polymerase (GenomiPhi V2 kit, GE Healthcare) according to the manufacturer’s instructions and used for Nextera DNA library construction (Illumina). Additionally, to evaluate the level of specimen cross-contamination that might occur after library construction [41], a uniquely indexed library of cDNA derived from the nematode Orsay virus RNA1 segment [42] was included in the pool for each sequencing run. Libraries were purified and size-selected using Agencourt Ampure XP beads (Beckman-Coulter), followed by quantification on a 2100 Bioanalyzer (Agilent Technologies).

### Virome analysis (read-based)

To control for sample extraction and cross-sample contamination, we included two types of negative controls: buffer-only controls that underwent the same extraction process, and PBS spiked with Orsay virus (a nodavirus of nematodes). Illumina MiSeq sequencing reads (2 × 250 bp) were processed through the VirusSeeker workflow (version 0.063) [43]. In brief, VirusSeeker follows a hierarchical workflow to perform a quality filter, subtract host sequences (human), identify candidate viral sequences through curated virus-only databases, then search sequentially against comprehensive databases to remove potential false positives (i.e., candidate viral sequences that have higher similarity to non-viral sequences). VirusSeeker takes a multi-step approach to remove bacterial sequences. Candidate viral sequences are queried against NCBI bacteria reference genomes to remove bacterial sequences. Second, VirusSeeker also performs three sequential BLAST searches (MegaBLAST against the complete NCBI NT database, BLASTn against the complete NT database, and BLASTx against the complete NCBI NR database) to remove potential false-positive viral sequences that have higher similarity to non-viral sequences (bacterial, fungal, etc.).

Contaminants were identified by decontam (version 1.0.0) [40] in a 2-step process, which uses a prevalence-based method to determine contaminant taxa. First, samples were compared to “Orsay” controls in decontam at a threshold of 0.1, and identified contaminant taxa were removed. Next, buffer controls were compared to samples using decontam at a stricter threshold of 0.5, and contaminants removed likewise. The resulting quality-controlled sequencing reads of each sample were then normalized to 31,000. Virus species with fewer than three reads were masked.

Ecological analyses (richness, alpha diversity—Shannon Index) were performed using Vegan R package (version 2.5-2) [44].The beta diversity and PCoA plots were obtained by using QIIME [34] using the Bray-Curtis distance on a presence-absence species data matrix. Results were plotted in GraphPad Prism (version 8) and the statistical significance was assessed using a non-parametric Mann-Whitney *U* test. For the familial relative abundance plot, only paired data of one mother and at least one infant per family were used.

### Metadata

#### MaAslin

MaAslin, a multivariate linear modeling tool, was utilized in finding the association between study metadata and relative abundance [45]. The significance of each association is given via a q-value; any relationship with a *q*-value of less than 0.05 is shown. For the metadata categories exclusive to infants such as, delivery route, feeding type, and zygosity, only the infant data was used. All sample data was used for the other general categories such as, mother/infant, delivery site, pre-pregnancy BMI [category], and race.

#### Random forest

RandomForest was used to classify metadata categories (version 4.6-14) [46]. Five hundred trees were built using randomForest and the Out-Of-Bag samples were used to test the classifier and obtain their error rates. The ROC curves and AUC measures for the classifiers were made by a R package, pROC, which plots pseudo-probabilities from classification tree votes for OOB samples (version 1.13.0) [47]. Similar to MaAslin metadata analysis, for the metadata categories exclusive to infants, such as, delivery route, feeding type, and zygosity, only the infant data was used. For the other general categories such as mother/infant, delivery site, pre-pregnancy BMI [category], and race, all sample data was used.

### Virome contig analyses

#### Contigs databases

Using IDBA_UD, the QC reads of each sample were used to create initial raw contigs (version 1.1.0) [48]. These contigs were then filtered using bbtools [31] to remove contigs with length 500 bp and de-duplicated at minidentity ≥ 99. The contigs created from buffer samples were queried against NT database using megablast and any contig with a coverage ≥ 95 and percent identity ≥ 97, was designated as a true contaminant and used to create a database of contaminant contigs. Megablast was used to query sample contigs against contig contaminant database and any hit to this database resulted in the removal of such contig from sample contigs. After contaminated contig removal, for each family, the contigs were concatenated into one file and then merged to create longer contigs using minimus2 [49]. These familial contigs were queried against viral NR database using blastx and the contigs with a hit against this database were used to create the familial viral contig database. We also corroborated these contig analyses by identifying bacteriophage contigs with VirSorter (parameters—virome, -db 1) (Additional file 4: Figure S4) [50].

#### Contig transmission

To identify viral contigs transmitted within each family group, BWA-MEM was used to map the QC reads of each sample to their corresponding familial viral contig database (version 0.7.17) [51]. The parameters -L 97,97 and -M were used to mark short reads as secondary. The mapped reads were used to measure shared contigs between mother and infant and used to determine potential transmission of virome from mother to infant. If a mother and one of her related infants had a read mapped to the same contig, then it was assumed to be shared between mother and infant; otherwise, it was exclusive to mothers or infants. Only families that had a sample from mother and at least one of her infants were used to determine potential mother to infant transmission. VirSorter-identified contigs were also analyzed in an identical manner, by generating a VirSorter contig database and mapping reads to the database to determine potential mother to infant transmission (Additional file 4: Figure S4).

### Transkingdom analysis

To characterize transkingdom interactions in mother-to-infant transmission, Pearson’s correlations were performed between bacterial ASVs and bacteriophage contigs that were shared between mothers and at least one of her infants. Bacterial ASVs and bacteriophage contigs that were present in only one family were excluded. Correlations were performed separately by delivery route and clustered on the vaginal delivery dataset using the R package gplots. To contrast differences in the C-section profile, the same clustering order (vaginal delivery) was used to visualize correlations in the C-section dataset. Pearson’s correlation was also performed between the ASVs and VirSorter identified bacteriophages.

### Statistics

Mann-Whitney *U* test was used to test significance when there were two independent groups, for example, related twins versus unrelated twins. Kruskal-Wallis test and Dunn’s multiple comparison were used to test significance between three or more groups, for example the feeding type of breastmilk, formula, or mixed. Pearson’s correlation was used obtain correlation between transmitted viruses and bacteria. All *p* values were two-tailed.

### Code availability

A fully reproducible workflow of the analysis presented in this manuscript can be found at https://github.com/RachelRodgers/Holtz-Lim_MotherTwinInfant_Virome_BacterialMicrobiome

## Supplementary information

**Additional file 1: Figure S1.** Metadata analysis of bacterial ASVs. (A) Multidimensional scaling (MDS) plot of the unweighted UniFrac distance with normal confidence ellipses. Expanded version shows eighteen samples encapsulated by the confidence ellipse of the buffer control samples (B) Output of MaAslin and random forest analysis. (C) Important ASVs and their statistical significance as assessed by MaAslin and random forest. (D) Heatmap of MaAslin identified ASVs for mothers and infants. (E) ROC curves and AUC measures for delivery route and mother/infant classification using random forest and pROC packages in R. Pseudo-probabilities are plotted on graph using Out-Of-Bag (OOB) sample tree classification votes. Only infant data used in vaginal vs. C-section classification while both mother and infant data used for mother vs. infant classification. (F) Weighted UniFrac pairwise comparisons. Statistical significance assessed by Mann-Whitney, and Kruskal-Wallis with Dunn’s multiple correction (feeding type: breastmilk vs. formula; breastmilk vs. mix; formula vs. mix). (G) Weighted UniFrac pairwise comparisons for related mother infant pairs. Statistical significance assessed by Kruskal-Wallis and Mann-Whitney.

**Additional file 2: Figure S2.** Relative abundance of the 10 most frequently shared (mother-infant) ASVs. Taxonomy shown at the genus level.

**Additional file 3: Figure S3.** Metadata analysis of viral species. (A) Number of sequencing reads in infant and maternal samples. Statistical significance assessed by Mann-Whitney. (B) Output of MaAslin and random forest analysis. (C) Important viral species and their statistical significance as assessed by MaAslin and random forest. (D) Heatmap of MaAslin identified viral species for mothers and infants. (E) ROC curves and AUC measures for delivery route and mother/infant classification using random forest and pROC packages in R. Pseudo-probabilities are plotted on graph using Out-Of-Bag (OOB) sample tree classification votes. Only infant data used in vaginal vs. C-section classification while both mother and infant data used for mother vs. infant classification. (F) Bray-Curtis Dissimilarity pairwise comparisons. Statistical significance assessed by Mann-Whitney. Kruskal-Wallis with Dunn’s multiple correction was used to assess feeding type (breastmilk vs. formula; breastmilk vs. mix; formula vs. mix) and Pre-pregnancy BMI (obese vs. overweight; obese vs. normal; overweight vs. normal). (G) Bray-Curtis Dissimilarity pairwise comparisons for related mother infant pairs. Statistical significance assessed by Mann-Whitney. Kruskal-Wallis with Dunn’s multiple correction was used to assess feeding type (breastmilk vs. formula; breastmilk vs. mix; formula vs. mix) and Pre-pregnancy BMI (obese vs. overweight; obese vs. normal; overweight vs. normal).

**Additional file 4: Figure S4.** VirSorter viral contig transmission analysis. Analyses of VirSorter contigs were performed as in Fig. 4. (A) The number of infant VirSorter viral contigs shared with their mother (top), and their proportion out of the total infant virome (VirSorter identified) is shown (bottom). Percent of infant VirSorter contigs that are shared with their mother is shown in blue and infant only VirSorter contigs are shown in green. (B) Average VirSorter contig proportion of mothers’ virome that is shared with infant or unique to the mother. (C) Average VirSorter contig proportion of infant virome that is shared with mother by delivery route. Statistical significance assessed by Mann-Whitney test. (D) Average VirSorter contig proportion of maternal virome that is shared with infant by delivery route. Statistical significance assessed by Mann-Whitney test. (E) Plot of the number of infant shared VirSorter viral contigs vs. the total number of maternal VirSorter contigs. Linear regression line fit to data is shown.

**Additional file 5: Figure S5.** Transkingdom interaction between bacteria and bacteriophage. Correlation of shared bacterial ASVs and viral contigs. (A) Heatmap shows the Pearson correlation between bacterial ASVs and bacteriophage contigs in infants from C-section delivery (left) clustered by hierarchical clustering. Second heatmap shows correlations from infants delivered vaginally clustered in the same order as correlations from infants delivered by C-section. B) Heatmap shows the Pearson correlation between bacterial ASVs and VirSorter identified bacteriophage contigs in infants delivered vaginally (left) clustered by hierarchical clustering. Second heatmap shows correlations from infants delivered by C-section clustered in the same order as correlations from infants delivered vaginally. These VirSorter analyses corroborate the contig analyses shown in Fig. 5.

**Additional file 6: Table S1.** Cohort metadata and sample inclusion status.

**Additional file 7: Table S2.** Read counts and taxonomy of ASVs shared by both twins.

**Additional file 8: Table S3.** Taxonomy of the 10 most frequently transmitted ASVs.

## Data Availability

Sequence data has been deposited to the European Nucleotide Archive under accession number PRJEB33578. Reads mapping to human have been removed from the submitted metagenomic sequence data. A fully reproducible workflow of the analysis presented in this manuscript can be found at https://github.com/RachelRodgers/Holtz-Lim_MotherTwinInfant_Virome_BacterialMicrobiome
