# Supplementary material for: Discordant transmission of bacteria and viruses from mothers to babies at birth
Source: Microbiome. 2019 Dec 10;7:156. doi: 10.1186/s40168-019-0766-7 (PMC6902606; doi:10.1186/s40168-019-0766-7)
Supplement: Supplementary file 2 — Additional file 1: Figure S1. Metadata analysis of bacterial ASVs. (A) Multidimensional scaling (MDS) plot of the unweighted UniFrac distance with normal confidence ellipses. Expanded version shows eighteen samples encapsulated by the confidence ellipse of the buffer control samples (B) Output of MaAslin and random forest analysis. (C) Important ASVs and their statistical significance as assessed by MaAslin and random forest. (D) Heatmap of MaAslin identified ASVs for mothers and infants. (E) ROC curves and AUC measures for delivery route and mother/infant classification using random forest and pROC packages in R. Pseudo-probabilities are plotted on graph using Out-Of-Bag (OOB) sample tree classification votes. Only infant data used in vaginal vs. C-section classification while both mother and infant data used for mother vs. infant classification. (F) Weighted UniFrac pairwise comparisons. Statistical significance assessed by Mann-Whitney, and Kruskal-Wallis with Dunn’s multiple correction (feeding type: breastmilk vs. formula; breastmilk vs. mix; formula vs. mix). (G) Weighted UniFrac pairwise comparisons for related mother infant pairs. Statistical significance assessed by Kruskal-Wallis and Mann-Whitney. [file 40168_2019_766_MOESM2_ESM.pdf]

Supplementary Figure 1

A

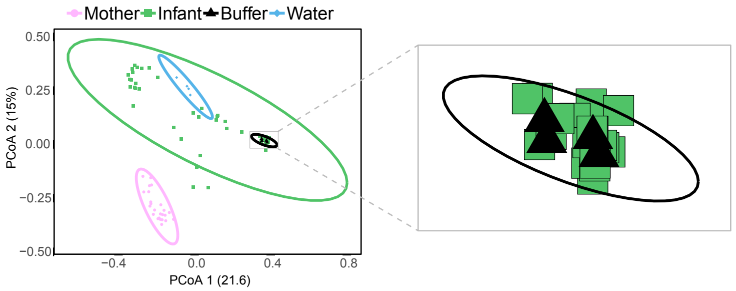

B

		MaAslin			Random Forest	
		Features	Average P.value	Average P.adjusted	OOB error rate (%)	AUC
Infant Microbiome	Zygosity	0	ns	ns	ns	ns
	Delivery Route	0	ns	ns	ns	ns
	Feeding Type	0	ns	ns	ns	ns
Mother & Infant Microbiome	Delivery Site	0	ns	ns	ns	ns
	Race	1	1.54E-06	6.93E-04	19.3	0.43
	Pre-preg BMI Cat	0	ns	ns	ns	ns
	Mother or Infant	**60**	**3.79E-05**	**7.66E-03**	**7.02**	**0.97**

C

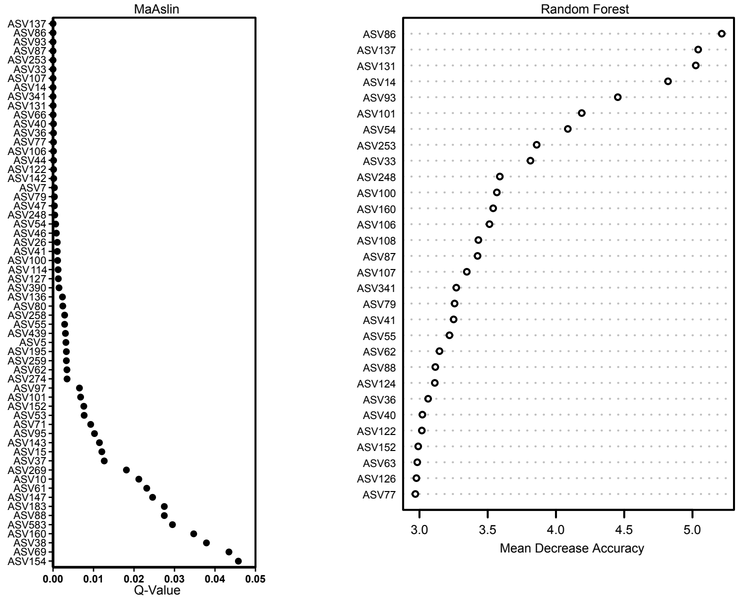

D

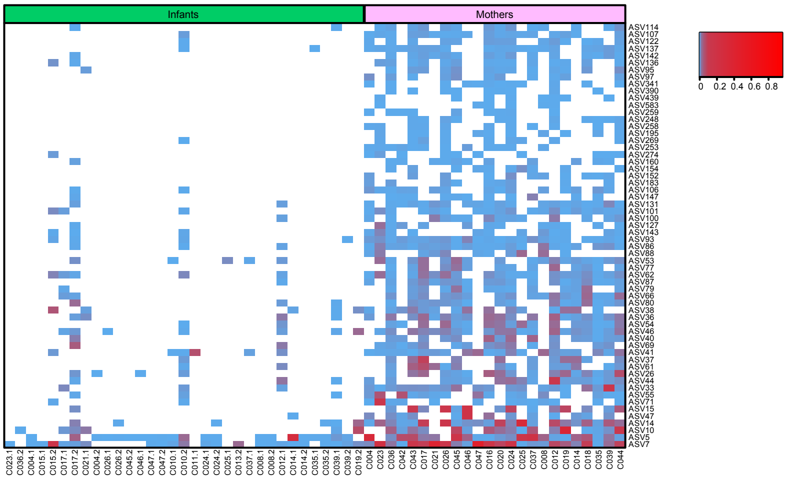

E

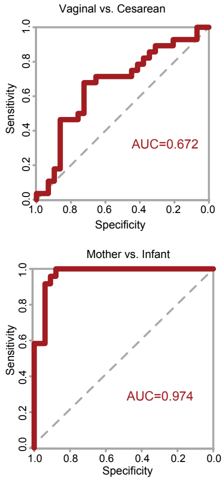

F

		Weighted Unifrac Distance
Infant Microbiome	Zygosity (related infants)	ns
	Delivery Route (all infants)	1.50E-05
	Feeding Type (all infants)	ns; ns; 0.002
Mother & Infant Microbiome	Delivery Site	6.22E-06
	Race	ns
	Pre-preg BMI Cat	ns
	Mother or Infant	2.10E-33

G

		Weighted Unifrac Distance
Related Mother Infant Paired Data	Zygostiy	ns
	Delivery Route	ns
	Feeding Type	ns
	Delivery Site	ns
	Race	ns
	Pre-preg BMI Cat	ns
	Mother or Infant	NA
