# Supplementary figures and images for: Discordant transmission of bacteria and viruses from mothers to babies at birth
Source: Microbiome. 2019 Dec 10;7:156. doi: 10.1186/s40168-019-0766-7 (PMC6902606; doi:10.1186/s40168-019-0766-7)

## Supplemental Figure 2

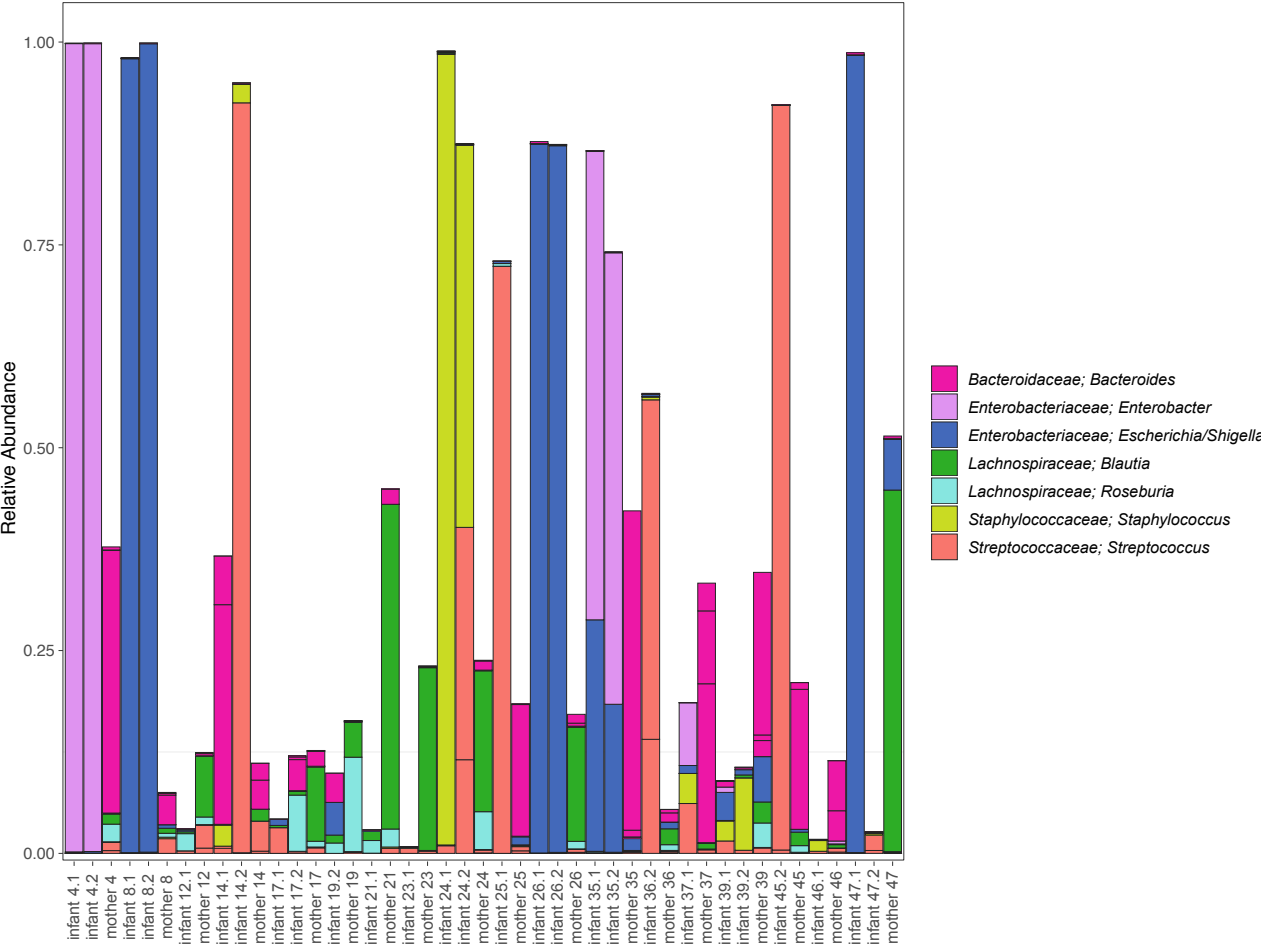

Supplement: Supplementary file 3 — Additional file 2: Figure S2. Relative abundance of the 10 most frequently shared (mother-infant) ASVs. Taxonomy shown at the genus level. [file 40168_2019_766_MOESM3_ESM.pdf]

Supplemental Figure 3

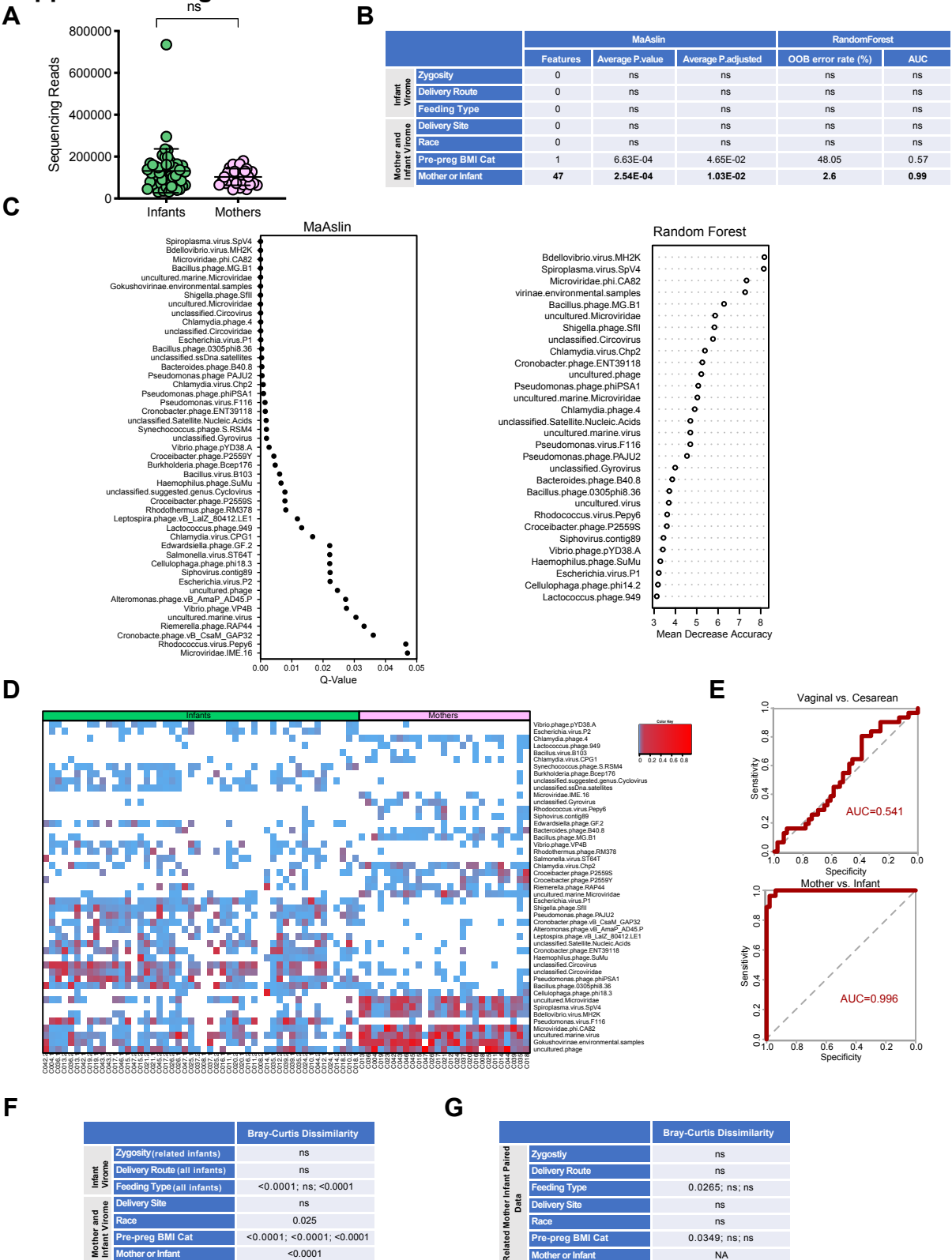

Supplement: Supplementary file 4 — Additional file 3: Figure S3. Metadata analysis of viral species. (A) Number of sequencing reads in infant and maternal samples. Statistical significance assessed by Mann-Whitney. (B) Output of MaAslin and random forest analysis. (C) Important viral species and their statistical significance as assessed by MaAslin and random forest. (D) Heatmap of MaAslin identified viral species for mothers and infants. (E) ROC curves and AUC measures for delivery route and mother/infant classification using random forest and pROC packages in R. Pseudo-probabilities are plotted on graph using Out-Of-Bag (OOB) sample tree classification votes. Only infant data used in vaginal vs. C-section classification while both mother and infant data used for mother vs. infant classification. (F) Bray-Curtis Dissimilarity pairwise comparisons. Statistical significance assessed by Mann-Whitney. Kruskal-Wallis with Dunn’s multiple correction was used to assess feeding type (breastmilk vs. formula; breastmilk vs. mix; formula vs. mix) and Pre-pregnancy BMI (obese vs. overweight; obese vs. normal; overweight vs. normal). (G) Bray-Curtis Dissimilarity pairwise comparisons for related mother infant pairs. Statistical significance assessed by Mann-Whitney. Kruskal-Wallis with Dunn’s multiple correction was used to assess feeding type (breastmilk vs. formula; breastmilk vs. mix; formula vs. mix) and Pre-pregnancy BMI (obese vs. overweight; obese vs. normal; overweight vs. normal). [file 40168_2019_766_MOESM4_ESM.pdf]

# Supplemental Figure 4

**A**

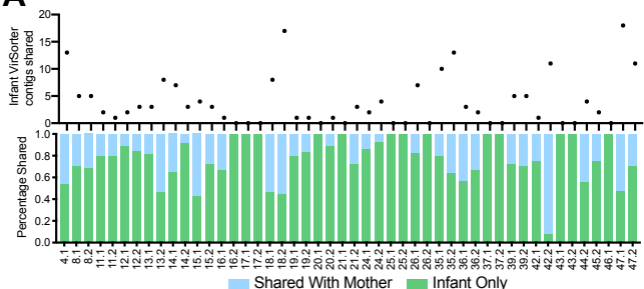

**B**

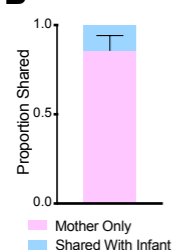

**C**

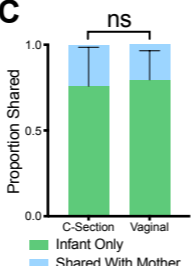

**D**

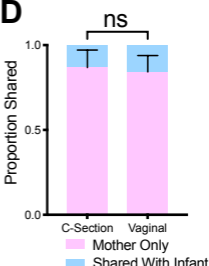

**E**

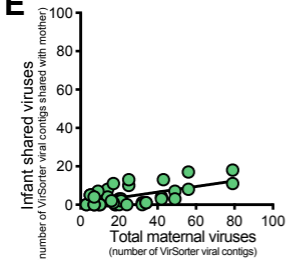

Supplement: Supplementary file 5 — Additional file 4: Figure S4. VirSorter viral contig transmission analysis. Analyses of VirSorter contigs were performed as in Fig. 4. (A) The number of infant VirSorter viral contigs shared with their mother (top), and their proportion out of the total infant virome (VirSorter identified) is shown (bottom). Percent of infant VirSorter contigs that are shared with their mother is shown in blue and infant only VirSorter contigs are shown in green. (B) Average VirSorter contig proportion of mothers’ virome that is shared with infant or unique to the mother. (C) Average VirSorter contig proportion of infant virome that is shared with mother by delivery route. Statistical significance assessed by Mann-Whitney test. (D) Average VirSorter contig proportion of maternal virome that is shared with infant by delivery route. Statistical significance assessed by Mann-Whitney test. (E) Plot of the number of infant shared VirSorter viral contigs vs. the total number of maternal VirSorter contigs. Linear regression line fit to data is shown. [file 40168_2019_766_MOESM5_ESM.pdf]

**Supplemental Figure 5**

**A**

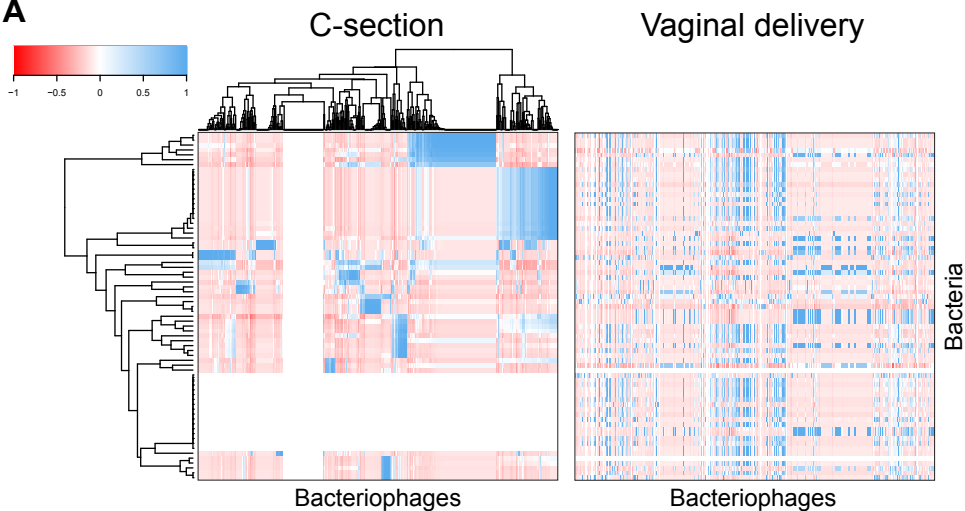

**B**

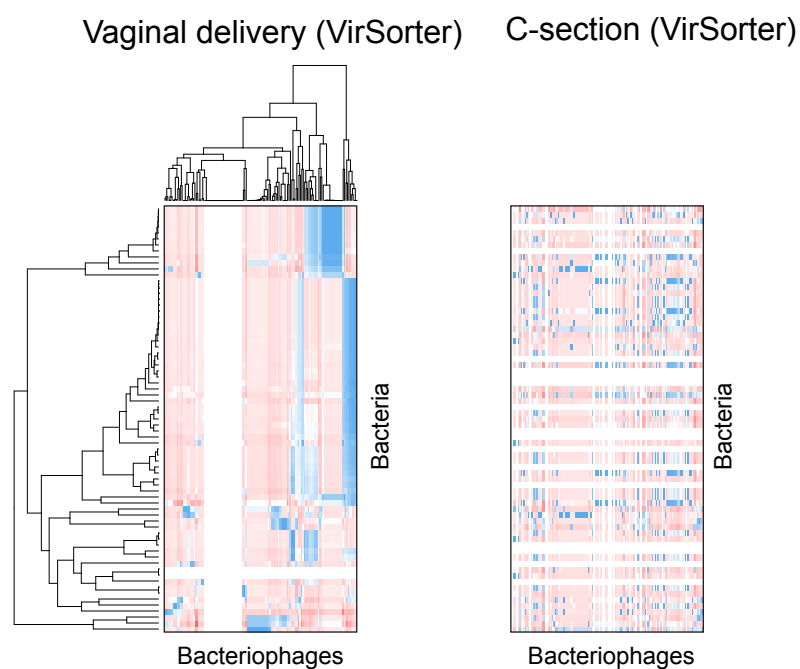

Supplement: Supplementary file 6 — Additional file 5: Figure S5. Transkingdom interaction between bacteria and bacteriophage. Correlation of shared bacterial ASVs and viral contigs. (A) Heatmap shows the Pearson correlation between bacterial ASVs and bacteriophage contigs in infants from C-section delivery (left) clustered by hierarchical clustering. Second heatmap shows correlations from infants delivered vaginally clustered in the same order as correlations from infants delivered by C-section. B) Heatmap shows the Pearson correlation between bacterial ASVs and VirSorter identified bacteriophage contigs in infants delivered vaginally (left) clustered by hierarchical clustering. Second heatmap shows correlations from infants delivered by C-section clustered in the same order as correlations from infants delivered vaginally. These VirSorter analyses corroborate the contig analyses shown in Fig. 5. [file 40168_2019_766_MOESM6_ESM.pdf]
